# Mixed-trophies biofilm cultivation in capillary reactors

**DOI:** 10.1016/j.mex.2019.07.021

**Published:** 2019-07-25

**Authors:** Ingeborg Heuschkel, Anna Hoschek, Andreas Schmid, Bruno Bühler, Rohan Karande, Katja Bühler

**Affiliations:** Department of Solar Materials, Helmholtz-Centre for Environmental Research, UFZ Permoserstrasse 15, 04318, Leipzig, Germany

**Keywords:** Photoautotrophic biofilm cultivation, *Synechocystis* sp. PCC 6803, Pseudomonas, Mixed-species biofilm, High cell density culture, Photosynthesis

## Abstract

The biocatalytic application of photoautotrophic organisms is a promising alternative for the production of biofuels and value-added compounds as they do not rely on carbohydrates as a source of carbon, electrons, and energy. Although the photoautotrophic organisms hold potential for the development of sustainable processes, suitable reactor concepts that allow high cell density (HCD) cultivation of photoautotrophic microorganisms are limited. Such reactors need a high surface to volume ratio to enhance light availability. Furthermore, the accumulation of high oxygen concentrations as a consequence of oxygenic photosynthesis, and its inhibitory effect on cell growth needs to be prevented. Here, we present a method for HCD cultivation of oxygenic phototrophs based on the co-cultivation of different trophies in a biofilm format to avoid high oxygen partial-pressure and attain HCDs of up to 51.8 g_BDW_ L^−1^ on a lab scale. In this article, we show:

• A robust method for mixed trophies biofilm cultivation in capillary reactors
• Set-up and operation of a biofilm capillary reactor
• A method to quantify oxygen in the continuous biofilm capillary reactor

## Graphical abstract

**Figure:**
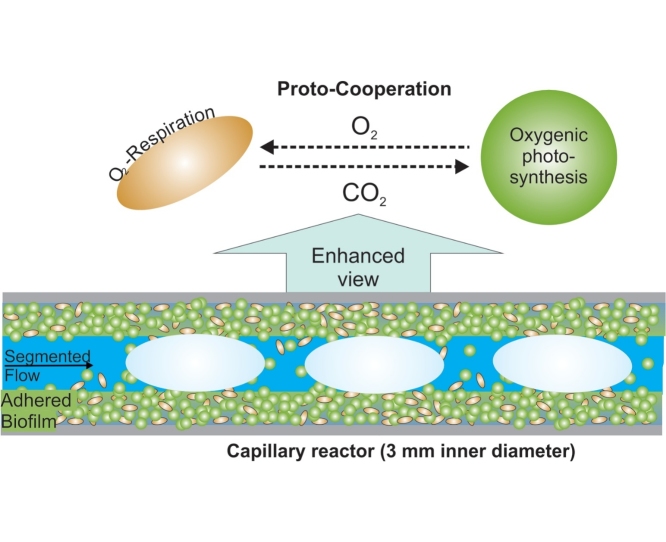

**Specifications Table**

**Table:** 

Subject Area:	Agricultural and Biological Sciences
More specific subject area:	Whole-cell (photo-)biotechnology based on biofilm catalysts
Method name:	Photoautotrophic biofilm cultivation
Name and reference of original method:	David, C., Bühler, K. & Schmid, A. J Ind Microbiol Biotechnol (2015) 42: 1083. https://doi.org/10.1007/s10295-015-1626-5 Hoschek,A., Heuschkel, I., Schmid, A., Bühler, B., Karande, R., Bühler K. Biores tech (2019) https://doi.org/10.1016/j.biortech.2019.02.093
Resource availability:	All resource information needed to reproduce this method is integrated in the paper (e.g., tubing, material and equipment).

## Method details

### Background

The application of microorganisms for biocatalytic purposes shows high value for the eco-efficient production of chemicals [1]. Chemoheterotrophic whole-cell biocatalysts rely on cheap and renewable organic carbon and energy sources such as glucose or citrate. Whereas, photoautotrophic biocatalysts exploit water as an electron source, use light as an energy source, and fix CO_2_ as an inorganic carbon source. Besides the development of catalytically efficient strains, the need for economically valuable reactor systems challenges the photo-bioprocess design [2]. Tubular photobioreactors are applied on a large scale for the production of high-value compounds [3].

Catalyst-coated capillary reactors show great promise due to the high surface area to volume ratio (1000–4000 m^2^ m^−3^) for the establishment of efficient continuous bioprocesses. Scaling of such a system is based preferably on the numbering-up approach [4]. For optimal light conversion, capillary reactors necessitate creative design concepts ensuring the optimal ground area to aerial surface ratio. Applying the microbial catalyst in the form of a biofilm further intensifies this technology, featuring self-immobilization, regeneration, and high biomass retention [5,6]. However, biofilm-based tubular microreactors are mainly restricted due to the fact that: *i)* not all microorganisms form (stable) biofilms in the capillary systems and *ii)* as a consequence of O_2_ respiration (heterotrophic organisms) or O_2_ evolution (phototrophic organisms), dense cultivation of microbes results in a microenvironment either being O_2_ limited or O_2_ overloaded, respectively [7,8].

Nature often provides solutions for such technological challenges. In microbial mats, for instance, oxygenic phototrophs and aerobic heterotrophs are embedded in a matrix of exopolysaccharides interacting in a symbiotic relationship [9]. In principle, this relationship relies on a microenvironment exploiting the complementary metabolic activities, inter alia, by the exchange of O_2_. Already in 1982, Adlerkreutz et al. exploited this *in situ* O_2_ exchange for biocatalytic purposes by the defined co-cultivation of the algae *Chlorella pyrenoidosa* and the bacterium *Gluconobacter oxydans* [10,11]. Several further studies exemplified the concept, e.g., for the lipid production by co-cultivating the oleaginous yeast *Rhodotorula glutinis* and the microalga *Chlorella vulgaris* [12]. These concepts focused on the exchange of metabolites using suspended or immobilized cells embedded in artificial polymers such as alginate [13]. We developed a method that takes up the concept of microbial mats in a defined and minimized system. Two microbial species with complementary metabolic activities are grown as a biofilm in a capillary reactor, featuring a stabilized cultivation system with enhanced biomass retention. The growth of single species phototrophic biofilms in capillary reactors is hampered by insufficient attachment and by oversaturated oxygen in the aqueous phase. Co-cultivation of the heterotrophic oxygen respiring strain *Pseudomonas taiwanensis* VLB120 with the photoautotrophic *Synechocystis* sp. PCC 6803 solved the problem of high oxygen concentrations and facilitated cell attachment and HCD biomass formation in the capillary reactor [14]. In the future, this method will be evaluated in the context of scaling a biofilm-based capillary reactor system. Additionally, sustainability aspects of the developed method to allow HCD will be compared to the conventional photobioreactors by using sustainability assessment tools like LCA as described in [15].

## Selection of the microbial strains

The photoautotrophic cyanobacterium *Synechocystis* sp. PCC 6803 was chosen as the model photoautotrophic organism as it is the current workhorse in the area of photo-biotechnology [16]. This strain was obtained from the Pasteur Culture Collection, PCC, Paris, France. As oxygen respiring organism, the heterotrophic *Pseudomonas taiwanensis* VLB120 was selected due to its excellent biofilm forming abilities and huge biocatalytic potential, which was already investigated in numerous studies [[17], [18], [19], [20]]. This strain was taken from our in house collection, and is also available at the German Collection of Microorganisms and Cell Cultures, DSMZ, Braunschweig, Germany under the reference number 26739.

1 Cultivation of the microorganisms in suspended culture
  (a) Cultivation of *Synechocystis* sp. PCC 6803 in suspended culture
    (i) For pre-culture, 20 mL YBG11 medium without citrate were inoculated with 200 μL of a *Synechocystis* sp. PCC 6803 cryo-stock in a 100 mL baffled shake flask closed with a cotton plug.
    (ii) Cultivation was performed at 30 °C, 50 μE m^-2^ s^-1^ (LED), ambient CO_2_ (0.04%), 150 rpm (2.5 cm amplitude), and 75% humidity in an orbital shaker (Multitron Proshaker, Infors, Bottmingen, Switzerland) for 4 days.
    (iii) From these pre-cultures main cultures in 20 mL YBG11 medium in 100 mL baffled shake flasks were inoculated to an OD_750_ of 0.08 and incubated for 3 days under the conditions given in (aii).
  (b) Cultivation of *Pseudomonas* sp. VLB120 in suspended culture
    (i) For pre-culture I, 5 mL LB medium were inoculated with an inoculation loop of a *Pseudomonas taiwanensis* VLB120 cryo stock in a 20 mL test tube.
    (ii) Cultivation was performed at 30 °C, 200 rpm (2.5 cm amplitude) in a Multitron Pro shaker (Infors) overnight.
    (iii) For preculture II, 20 mL M9 medium (5 g L^-1^ citrate, US* trace elements) were inoculated with 200 μL of preculture I and in 100 mL baffled shake flasks and cultivated for 24 h under the same conditions as given in (bii).
    (iv) For the main culture, 20 mL M9 medium (5 g L^-1^ citrate, US* trace elements) were inoculated from preculture II to an OD_450_ of 0.2 and incubated in 100 mL baffled shake flasks for 8 h under the same cultivation conditions as given in (bii).
  (c) Cultivation of mixed trophies in suspended culture
    (i) Respective main cultures containing either *Synechocystis* sp. PCC 6803 or *Pseudomonas taiwanensis* VLB120 were centrifuged (5000 g, RT, 7 min) and the pellets were re-suspended in 2 mL YBG11 medium (w/o citrate, 50 mM NaHCO_3_). 20 mL YBG11 medium (w/o citrate, 50 mM NaHCO_3_) were inoculated from both cell suspensions with OD_750_ for the *Synechocystis* sp. culture and OD_450_ for the *Pseudomonas* sp. culture of 1, and incubated in a 100 mL baffled shake flask for 24 h under the conditions given in (aii).
2 Capillary reactor assembly

**Fig. 1:**
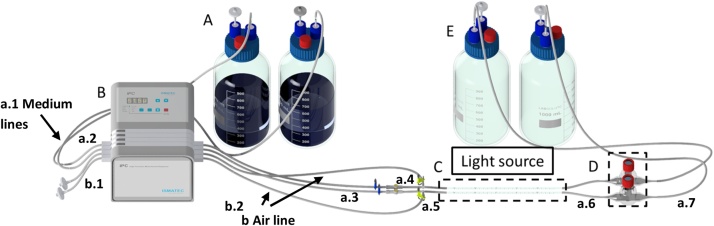
Set-up of the biofilm capillary reactor system. (**A**) Medium bottles, (**B**) multichannel peristaltic pump, (**C**) cultivation unit, (**D**) bubble trap, (**E**) Waste reservoir. Small letter indices refer to the different tubings and are specified in Table 1.

Fig. 1 shows the overall capillary reactor set-up. Table 1 lists all parts in the required specification.

(a) Preparation of the feed and waste bottles

**Table 1:** Parts necessary for the assembly of 2 capillary reactor units, as shown in Fig. 1.

Part	Material	Length	Inner diameter	Amount
Medium lines				
a.1	Tygon	20 cm	2 mm	2x
a.2 (Pump tube)	Tygon	Pre-cut	2 mm	2x
a.3	Tygon	10 cm	2 mm	2x
a.4	Tygon	1 cm	2 mm	2x

Connection to the cultivation unit				
a.5	Silicon	2 cm	2 mm	2x
a.6	Silicon	2 cm	2 mm	2x

Waste line				
a.7	Tygon	35 cm	2 mm	2x

Aeration lines				
b.1 (Pump tube)	Tygon	Pre-cut	2 mm	2x
b.2	Tygon	10 cm	2 mm	2x

Medium bottle (Fig. 2)				
mb.1	PTFE	30 cm	2 mm	2x
mb.2	Silicon	2 cm	2 mm	2x

Waste bottle				
wb.1	PTFE	6 cm	2 mm	2x
wb.2	Silicon	2 cm	2 mm	2x

Part	Material^a^	Size	Supplier	Product identifier	Amount
Inline sample port (Fig. 6)	Glass				2x
Puradisc Syringe Filter	PES	0.2 μm, 25mm	Whatman	6759-2502	2x
Serological Pipette	PS	1 mL	Starstedt	86.1251.001	2x
In-line Luer injection ports (Fig. 3)	PC		ibidi	10820	2x
IPC 4 peristaltic pump		4 channel	Ismatec	ISM 930	1x
Tubing connectors	PP	2.5 mm, T-shape	Ismatec	ISM 557	2x
	PP	2.5 mm, straight	Ismatec	ISM 694	12x
	PP	male Luer lock to barb connector	Qosina	11399	2x
	PP	female Luer lock to barb connector	Qosina	11534	2x
Screw cap		GL 45, 3 port x GL 14	Duran	11 297 51	2x
		GL 14 for hose connection	Duran	11 298 14	4x
Insert for screw cap		ID 3.0 mm OD for GL14	Duran	11 298 16	4x
Pressure compensation set		GL14, 0.2 μm filter	Duran	11 377 99	2x
Laboratory bottle		2 L, GL 45	Duran	21 801 54 14	2x

a PES: polyetheresulfone membrane, PS: polystyrene, PC: polycarbonate; PP: polypropylene.

It is recommended to use one 2 L bottle for the feed and another one as a waste reservoir for two simultaneously operated capillaries.

**Fig. 2:**
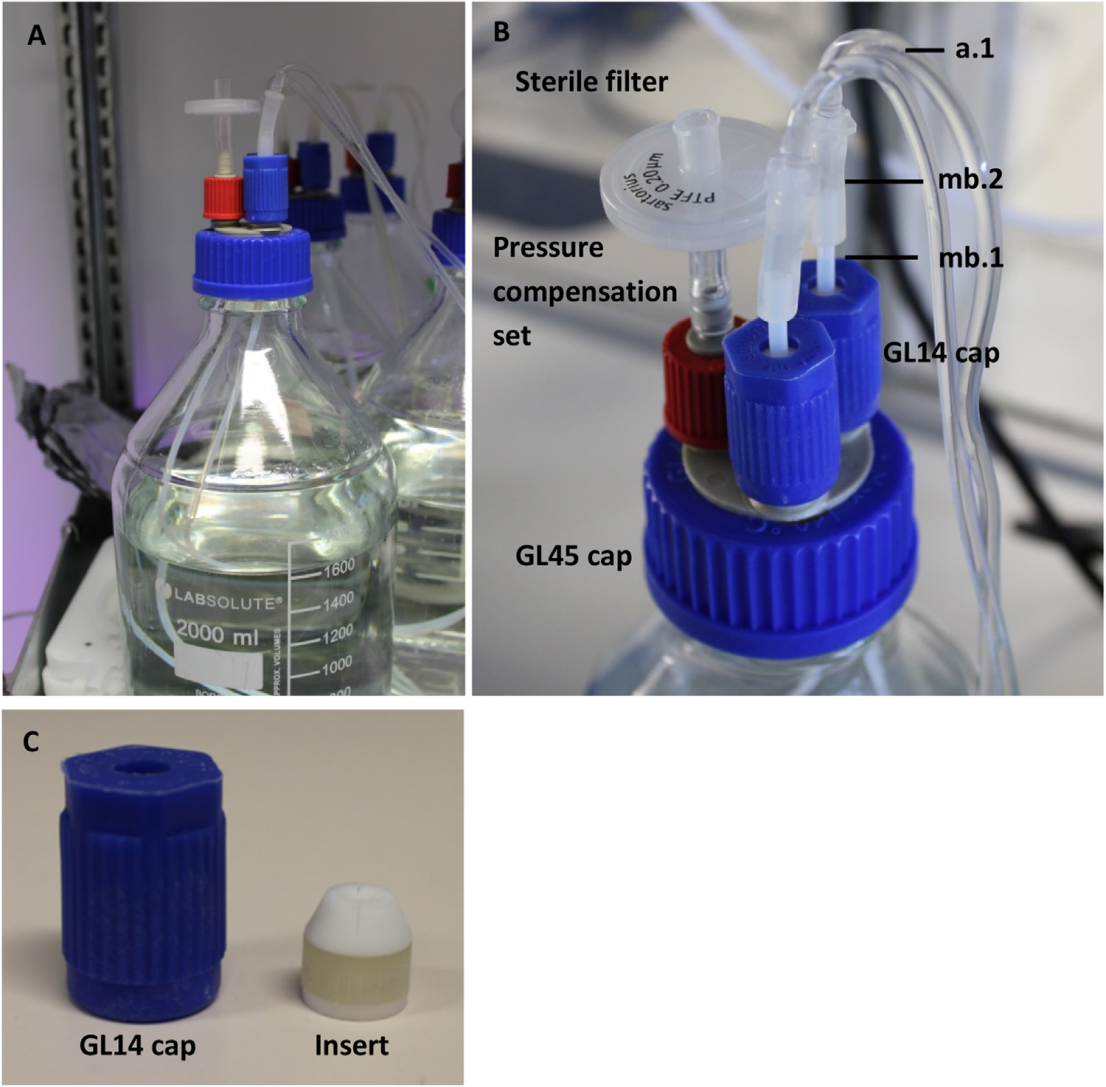
Medium bottle with screw cap and tubing. The medium bottle contained YBG11 which was fed into the capillary reactor. This setting as shown in Fig. 2 can supply two cultivation units. (A) Medium bottle with a fully assembled GL45 cap. (B) Close-up of the GL45 cap. (C) GL14 cap with the respective insert.
(ii) The medium bottle GL 14 cap was assembled with the respective insert as shown in figure 2C. Subsequently, PTFE tubes (**mb.1**, Table 1) were fitted to the cap, ending approximately 1.5 cm over the GL 14 cap as indicated in figure 2 AB. ***mb.1*** was fixed by closing the cap tightly.
(iii) The bottom side of ***mb.2*** was connected to ***mb.1*** and the top side to a straight tube connector (ISM 694). The other end of the straight tube connector was used connect ***a.1*** (described in section b).
(iv) For the preparation of the waste bottle, ***wb.1*** and ***wb.2*** were assembled according to the description given in (aii and aiii)
(v) Before autoclaving, the GL14 screw caps for hose connection were opened a little to prevent breakage, and the top of both bottles were wrapped in aluminum foil. **Note:** To avoid damaging the PFTE tubes during autoclaving, tubing needs to be autoclaved dry and straight (not curled up), which is easily accomplished by screwing the GL45 cap onto an empty bottle as described above. Alternatively, the whole cap and tubing may be wrapped in aluminum foil.
(vi) YBG11 medium was prepared according to [21] and sterilized via filtration.
(vii) After sterilization, tubing and cap were transferred to the medium bottle under sterile conditions.

(i) GL 45 cap, 3 port x GL 14 caps equipped with a pressure compensation set and sterile filter were put onto empty 1 L bottles (Fig. 2).

b Assembling the capillary reactor system

dfhfgh

In the following section, the procedure for the setup of two capillary reactor units will be described, both supplied with medium from the same reservoir and connected to the same pump. The reactors can be operated in single-phase mode, only pumping medium through the cultivation unit, or in two-phase mode pumping liquid medium and an air phase simultaneously (aqueous-air segmented flow).

**Fig. 3:**
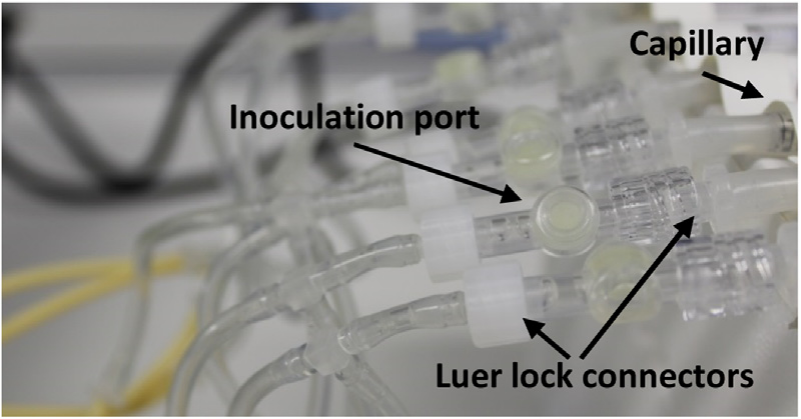
Assembled injection ports. The injection ports were obtained from ibidi (Martinsried, Germany) and attached to the medium line by via Luer lock tube connectors. Injection ports may be inserted before or after the air-line connection into the system. Here, the first option is described.
(viii) The other end of the inoculation tube was connected to ***a.4***
(ix) ***a.4*** was connected to a T-connector
(x) ***b.1*** was connected to a sterile filter at the one end and ***b.2*** via a straight tube connector at the other end
(xi) ***b.2*** was connected to the same T-connector mentioned in (ix). As the airline is only used for the segmented flow, it should be closed with a clamp during single phase medium flow.
(xii) For the cultivation unit (capillary), a sterile 1 mL pipette was cut to a specific length of 20 cm and connected to the T-connector mentioned in (ix) by ***a.5***. Various capillary materials (glass, PVC, silicon, etc.,) can be evaluated as cultivation units.
(xiii) The cultivation unit was connected to **a.6** and via a straight tube connector to ***a.7***.
(xiv) ***a.7*** is finally connected to the already assembled waste bottle via the straight tube connector on ***wb.2***.
(xv) 3Operational conditions of the capillary reactor
  (a) Inoculation of the capillary reactor
    (i) Before inoculation, the capillary reactor was conditioned for 2 h with the medium at a flow rate of 52 μL min^-1^.
    (ii) For inoculation, the flow was stopped, and the tubes closed by clamps at the inlet of the injection port. 2 mL of the mixed species cell suspension (1 ci) was purged into the system.
    (iii) The cells were allowed to settle/attach to the surface for 24 h without flow. During this time, the cultivation unit was kept in the dark to prevent photosynthesis and thus, oxygen evolution.
    (iv) After an attachment period of 24 h, the tubing at the inlet of the cultivation unit was opened again and the medium flow was re-started at a flow rate of 52 μL min^−1^ and the light source located on top of the cultivation unit was set to 50 μE m^-2^ s^−1^ (intensity measured right above the cultivation/capillary unit).
  (b) Cultivation under segmented flow conditions
    (i) The single-phase medium flow was continued until the cells in the cultivation unit started to turn yellow, indicating an oxygen toxification (Fig. 4B).

(i) Tubing ***a.1, a.2, a.3, a.4, a.5, a.6, a.7, b.1,*** and ***b.2*** were prepared according to Table 1.
(ii) All tubes were wrapped in aluminum foil for autoclaving. **Note:** Tygon tubing gets sticky during autoclaving, so a limited number of tubes (8-10) should be autoclaved together.
(iii) The appropriate number of connectors were prepared according to Table 1 and wrapped in aluminum foil for autoclaving
(iv) After autoclaving, all parts were assembled under the clean bench.
(v) ***a.1*** was connected at the one end to the medium bottle (straight tube connector, **mb.2**) and at the other end via a straight tube connector to the pump tube ***a.2***
(vi) ***a.2*** was connected via a straight tube connector to ***a.3***
(vii) ***a.3*** was connected to the injection port via Luer lock connectors (Fig. 3)

**Fig. 4:**
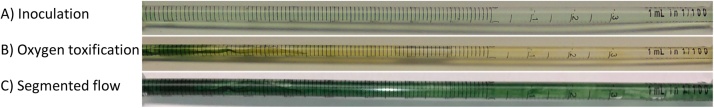
Three stages of biofilm development. A) Cultivation unit after 24 h of inoculation. B) *Synechocystis* sp. is turning yellow after approximately 11 days due to oxygen toxification. C) Cultivation unit after 48 h under segmented flow conditions. Oxygen is removed by the segments, and growth of *Synechocystis* sp. is promoted.
    (ii) To relive the oxygen toxification and reduce the dissolved oxygen tension air segments were fed into the cultivation unit by opening the airline. The air flow rate was equal to the medium flow rate at 52 μL min^-1^. Introduction of the air segments facilitated oxygen removal from the cultivation unit.
    (iii) The longer the biofilm was cultivated, the more biomass was formed. Fig. 5 shows a biofilm development for 34 days.

**Fig. 5:**
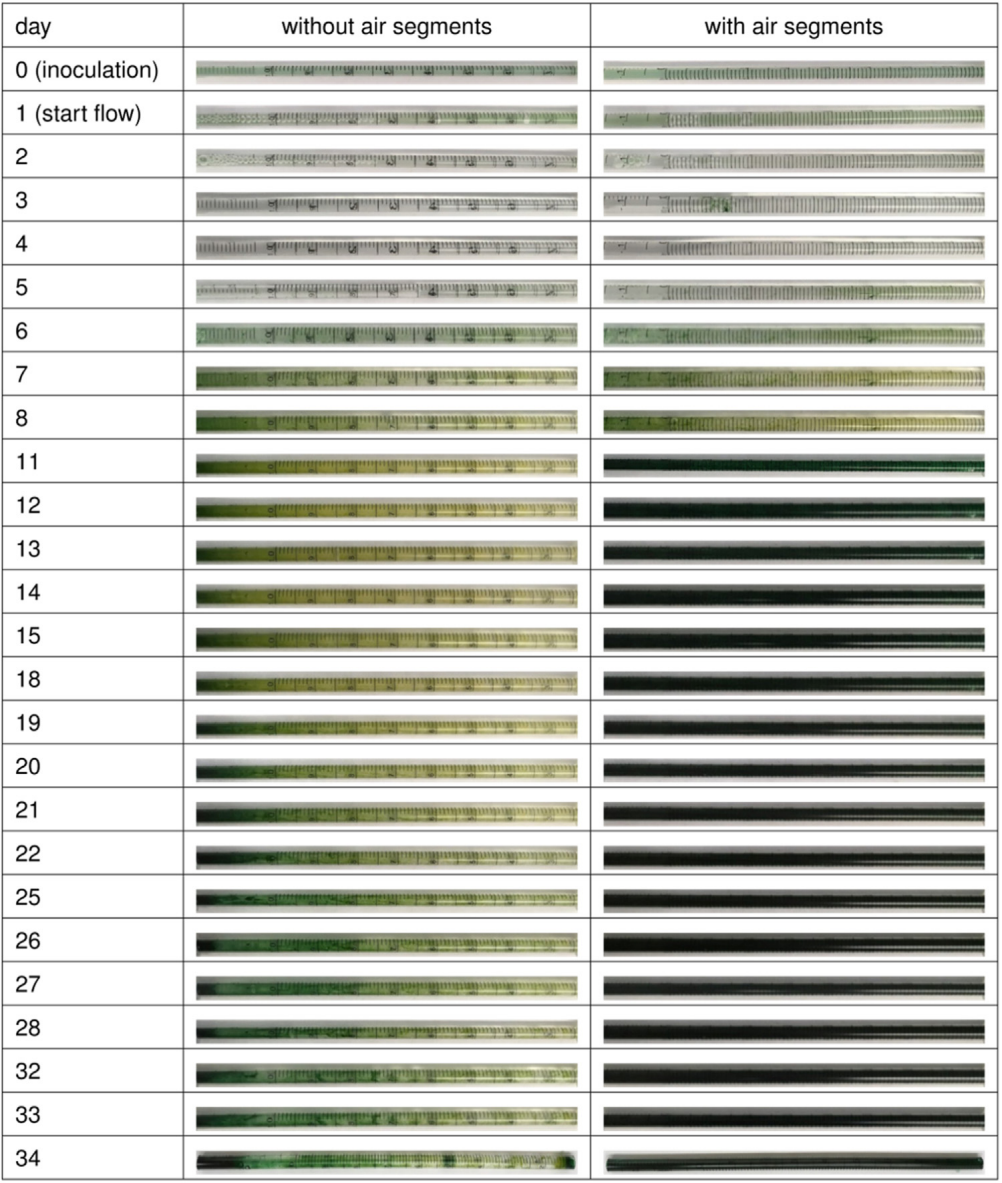
Image series of mixed trophies biofilm cultivation of *Synechocystis* sp. PCC 6803 and *P. taiwanensis* VLB 120 for 34 days. The biofilm was cultivated in YBG11 medium without addition of an organic carbon source. Cultivation procedure, as described in subchapter 3.
(xvi) 4Oxygen measurements in the capillary reactor
  (a) Measurement of dissolved oxygen

Dissolved oxygen in the aqueous phase was quantified by Clark-type oxygen microsensors using a flow cell attached to the microsensor OX-500, which was obtained from Unisense, Aarhus, Denmark. Clark-type sensors measure the oxygen partial pressure due to the diffusion of oxygen through a silicone membrane to an oxygen-reducing cathode, which is polarized against an internal Ag/AgCl anode. The measured current is recorded by a high-quality picoammeter (Microsensor multimeter, Unisense).

**Fig. 6:**
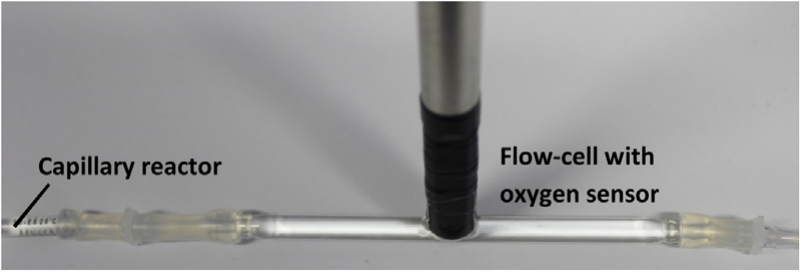
A flow cell with oxygen sensor connected to a capillary reactor.
    (iv) After the measurement the flow cell was rinsed with 96% ethanol, followed by 0.01 M HCl and finally flushed with water. Alternatively, it is possible to rinse it with 0.1 M NaOH or isopropanol.
    (v) The sensors were stored with the tip exposed either to water or air. It was possible to store it polarized connected to an amplifier for up to 5 days.
  (ii) Measurement of oxygen in the gas phase of the capillary reactor under segmented flow
    (i) For O2 quantification during segmented flow, custom made bubble traps as depicted in Fig. 7 were autoclaved and connected either to the inlet or the outlet of the cultivation unit. It was important that the silicon sealing was properly inserted as the cap needed to be tightly closed.

(i)
  (i)
    (i) The sensor was polarized and connected to the amplifier until it gave a steady signal.
    (ii) The sensor was calibrated according to the distributer’s manual before every use. It is advised to note down each value in mV and μmol L^-1^ for each measured value.
    (iii) The flow cell was connected either to the inlet or the outlet of the cultivation unit of the flow through the reactor as indicated in Fig. 6 and filled with medium. It was important that no bubbles accumulated in the flow cell as this would disturb the microsensor measurements.

**Fig. 7:**
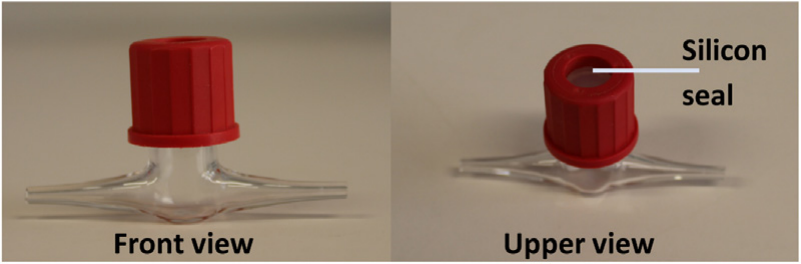
Custom made bubble trap for analyzing the gas phase of the capillary reactor.
    (ii) The bubble trap system was equilibrated for 24 h in the segmented flow before sampling the gas phase.
    (iii) A gas-tight syringe (Hamilton, Reno, NV) was inserted into the bubble trap via the silicon seal and flushed 1–2 times.
    (iv) Subsequently, 100 μL gas phase was withdrawn, manually injected, and analyzed by gas chromatography.
    (v) After using the gas-tight syringes was cleaned by rinsing it first with water followed by acetone.
